# Screening, Expression, Purification and Functional Characterization of Novel Antimicrobial Peptide Genes from *Hermetia illucens* (L.)

**DOI:** 10.1371/journal.pone.0169582

**Published:** 2017-01-05

**Authors:** Osama Elhag, Dingzhong Zhou, Qi Song, Abdul Aziz Soomro, Minmin Cai, Longyu Zheng, Ziniu Yu, Jibin Zhang

**Affiliations:** 1 State Key Laboratory of Agricultural Microbiology, National Engineering Research Center of Microbial Pesticides, College of Life Science and Technology, Huazhong Agricultural University- Wuhan, China; 2 Faculty of Science and Technology, Omdurman Islamic University, Khartoum, Sudan; Instituto Nacional de Salud Pública, MEXICO

## Abstract

Antimicrobial peptides from a wide spectrum of insects possess potent microbicidal properties against microbial-related diseases. In this study, seven new gene fragments of three types of antimicrobial peptides were obtained from *Hermetia illucens* (L), and were named cecropinZ1, sarcotoxin1, sarcotoxin (2a), sarcotoxin (2b), sarcotoxin3, stomoxynZH1, and stomoxynZH1(a). Among these genes, a 189-basepair gene (stomoxynZH1) was cloned into the pET32a expression vector and expressed in the *Escherichia coli* as a fusion protein with thioredoxin. Results show that Trx-stomoxynZH1 exhibits diverse inhibitory activity on various pathogens, including Gram-positive bacterium *Staphylococcus aureus*, Gram-negative bacterium *Escherichia coli*, fungus *Rhizoctonia solani* Khün (rice)-10, and fungus *Sclerotinia sclerotiorum* (Lib.) de Bary-14. The minimum inhibitory concentration of Trx-stomoxynZH1 is higher against Gram-positive bacteria than against Gram-negative bacteria but similar between the fungal strains. These results indicate that *H*. *illucens* (L.) could provide a rich source for the discovery of novel antimicrobial peptides. Importantly, stomoxynZH1 displays a potential benefit in controlling antibiotic-resistant pathogens.

## Introduction

The need for safe and effective antimicrobial peptides (AMP) has increased with the emergence of antibiotic-resistant strains due to excessive use of conventional antibiotics [1]. AMPs are a key factor of the innate immune system of many organisms and play an important role in host-protecting mechanisms from pathogen invasion [2, 3]. Different AMP databases facilitate the screening, identification, and characterization of new antimicrobial peptides [4]. So far, more than 2000 AMPs from vertebrates and invertebrates have been reported according to the antimicrobial peptide database [5].

Although insects lack the specific immune system found in higher animals, they have developed an effective and complex innate immune system obliviously different from the adaptive system of vertebrates [6, 7]. The quick and intensive production of potent AMPs is the main factor contributing to the defense weapons generated by insects to eliminate invading pathogens rapidly [8]. Several studies have focused on identifying AMPs and antimicrobial proteins from insects [5, 9, 10].

The black soldier fly *Hermetia illucens* (L.) of the order Diptera and family Stratiomyidae is a non-pest insect in tropical and warm-temperate regions that helps reduce large concentrations of animal manure and other bio-solids [11–13]. As an ecological decomposer, black solider fly is often exposed to relatively high concentrations of harmful microorganisms, such as bacteria and fungi [14]. Therefore, the survival of this insect is mainly depends on its development of an effective innate immune system that confers protection against microbial infections.

A considerable reduction was observed in *Escherichia coli* and *Salmonella enterica* when *H*. *illucens* (L.) consumed chicken manure [15]. Moreover, Liu et al. [16] reported that black soldier fly larvae can eliminate *E*. *coli* in dairy manure. A previous study found that larval extracts from the black soldier fly exhibit potential antimicrobial activities against *Staphylococcus aureus*, a methicillin-resistant *S*. *aureus*, and Gram-negative *Pseudomonas aeruginosa* [14]. Park et al. [17] have recently identified a new AMP of 40 amino acids and named it as defensin-like peptide4; this AMP was constitutively induced and exclusively purified from the immunized hemolymph of *H*. *illucens* larvae. Interestingly, this AMP exhibits bactericidal activity to Gram-positive bacterial strains. Therefore, elucidation of AMPs or their homologs could provide valuable data to understand the mechanism underlying innate immunity in *H*. *illucens* (L.). Consequently, these AMPs may potentially be a source of novel antibiotic-like compounds for antibiotic-resistant strains.

This study aimed to identify the AMPs of *H*. *illucens* (L.). The mature peptide of stomoxynZH1 was cloned and expressed in *E*. *coli* to obtain large quantities of the recombinant Trx-stomoxynZH1, and the antimicrobial activity of the recombinant peptide targeting different pathogenic organisms was examined.

## Materials and Methods

### Strains, plasmids, and reagents

*E*. *coli* DH5α, *S*. *aureus*, *Rhizoctonia*. *solani* Khün (rice)-10 and *Sclerotinia*. *sclerotiorum* (Lib.) de Bary-14., were obtained from the state key laboratory of agricultural microbiology of Huazhong Agricultural University, Wuhan-China. *E*. *coli* BL21 (DE3) and expression plasmid pET32a were purchased from Novagen (Piscataway, NJ, USA). TA cloning vector pMD18-T, T4 DNA ligase, and Taq DNA polymerase were purchased from TaKaRa Biotechnology (TaKaRa, Japan). AxyPrep DNA Gel Extraction Kit is the product of Axygen Scientific Inc (USA). TRIzol reagent and TransScript^™^III first-Strand cDNA Synthesis SuperMix were obtained from Invitrogen (USA).

### Insects, immunization, and total RNA extraction

Fifth instar *H*. *illucens* (L.) larvae of Wuhan strain reared on an artificial diet at 28°C and 65% humidity were obtained from a colony maintained year-round in a greenhouse [18] at the State Key Laboratory of Agricultural Microbiology, Huazhong Agricultural University-Wuhan-China. The larvae were first soaked in 75% alcohol for 30 min and then washed three times with sterile water. Each larva was deeply pricked with a fine needle dipped into the suspension of *S*. *aureus* and *E*. *coli* (OD600 = 1.5). At 1 day post injection, immunized larvae were crushed in an RNAase free mortar after treatment with liquid nitrogen. The total RNA was extracted using Trizol (Invitrogen) in accordance with the manufacturer’s protocol and then dissolved in water treated with diethylpyrocarbonate.

### First-strand cDNA production and cloning of the AMP genes

First-strand cDNA was synthesized with a first-strand synthesis kit (Invitrogen) and used for polymerase chain reaction (PCR). A total of 37 pairs of degenerate primers (Tables 1 and 2) were designed on the basis of the conserved sequences of the insects’ AMPs. A gradient PCR reaction was performed with 2 μL of first-strand cDNA template (50 ng/ μL) prepared in 50 μL volumes which containing 34.5 μL of ddH_2_O, 5.0 μL of 10× PCR buffer, 4.0 μL of dNTP mix (2.5 mM), 2 μL of each sense and antisense primer (10 μM), and 0.5 μL of Taq DNA polymerase (1 U/μL). The cycling parameters used were as follows: 94°C for 5 min; 30 cycles of 94°C for 30 s, 40–60°C for 20 s and 72°C for 30 s; and 72°C for 10 min. The amplified DNA products were electrophoretically analyzed by size fraction on agarose gels (2%) and then stained with ethidium bromide. The amplified fragments were recovered from the gel using the AxyPrep DNA Gel Extraction Kit and then cloned into a pMD18-T plasmid. Cloning and propagation of the recombinant plasmids were carried out in DH5α cells. For colony PCR, transformants were initially grown on LB plates supplemented with 100 μg/mL ampicillin and then 10 resistant colonies were selected to confirm the integration of the amplified fragment into the pMD18-T plasmid. The positive clones were sequenced by TSINGKE Biological Technology Company (Wuhan, China).

**Table 1:** Primers used for anti-bacterial peptide synthesis.

Primer	Sequence (5′-3′)
Attacin-F	MGWMGWCAAGTTTTRGGAGG
Attacin-R	WSWYAATCCYAATCCAG
Cecropin-F	ATGAACTTYTATAANATTTTCG
Cecropin-R	TCCWCKAGCAGTAGCAGCAA
Defensin-F	ATGAARTTYTTYGTTTTRGT
Defensin-R	RTTWCKRCAAACRCAAAGC
Lucifensin-F	GCTACTTGTGATTTRTTRTCWGGAA
Lucifensin-R	RTTWCKACAAACACAAATAGCWC
GmoDef-F	GTTACTTGTAAYATTGGAG
GmoDef-R	RTTAGTACAATAACAAACTCCWCKW
Drosocin-F	GGAAARCCTMGWCCTTAT
Drosocin-R	AACWCKAATAGGWCKAGGATGWG
Andropin-F	GTTTTYATTCATATTTTRC
Andropin-R	YTTAGGNWWAATYAWYTTTTCAATA
Cecropin1-F	GGAGGATTRAARAARTTRGGAAARA
Cecropin1-R	TCCHAWAGCYTTAWWTCCA
Bactrocerin-F	GTTGGAAARACTTGGATTA
Bactrocerin-R	TGCCAYTTAATYTTWGAYTTTCC
DiptericinM-F	ATGAARTATTTRTGKGCTATTG
DiptericinM-R	CCAWCKATAAGTATAAATTCCTCCT
DiptericinD-F	ATGCAATTYACTATTGCTGTTGC
DiptericinD-R	RAARTTAGGRAAWCKATAAG
DefensinM-F	GTTGCTGTTTGTYATATTTCWC
DefensinM-R	TCCYTTTCCRTTACAATATCCTCCW
DefensinD-F	ATGAARTTYTTYGTTTTRGTTGC
DefensinD-R	RTTWCKACAAACACAAACAGCYTTA
CecropinM-F	ATGAAYTTYAAYAARTTRTTYG
CecropinM-R	TCCYTTYAAAGTAGCAGCAACRTTA
CecropinA/B/C-F	GTTTTYGTTGCTTTRATTTTRGC
CecropinA/B/C-R	TCCWCKAGCAGTAGCAGCAACRTTA
AttacinM-F	ATGTTYWCWAARTCWAATG
AttacinM-R	RAAATCATGWGAYAATCCAACTCCA
Attacin C-D-F	ATGTCWAARAATGTTTTRTTRAATG
Attacin C-D-R	TCCRAAATAATGWGAYAATCCYA
Attacin B-C-F	MGWMGWCAAGTTTTRGGAGGAT
Attacin B-C-R	RAAAGGTCCWGAAGTCCAYTTWGAA
JyAttacin-D-F	ATGGAATGTCAAGCTTCWGGAAAYC
JyAttacin-D-R	TAWCKYAARTTAACTCCAGTTCCRA
Cecropint-F	GGATGGTTRAARAARATTGGAAARA
Cecropint-R	TCTAGCAGTAGCAGCAACRTTAGCA
Sapecin-F	GCTACTTGTCATTTRTTRTCWGGAA
Sapecin-R	RTTTCTACAAACACAAAYWSCYT
Sarcotoxin-F	GGATGGTTRAARAGAAARATTGGAA
Sarcotoxin-R	TCTAGCAGTAGCAGCAACRTTAGCA
Ceratotoxin-F	TCWATTGGATCWGCTTT
Ceratotoxin-R	AGCAGCYTTAGCAATAGGYAAAGCA
AaeDef-F	GCTACTTGTCATTTRTTRTCWGGAT
AaeDef-R	RTTTCTACAAACACAAACYTTYTTW
Smd-F	ACTTGTCATTTRTTRTCWATGTGGA
Smd-R	ACAAACACAHAMWSCWT

Letters Y, R, W, S, M, N, K, and H in degenerate primers represent nucleotide mixtures of C/T, A/G, A/T, G/C, A/C, A/C/G/T, G/T, and A/C/T, respectively.

**Table 2:** Primers used for anti-fungal peptide synthesis.

Primer	Sequence (5′-3′)
AgaDef-F	GCTACTTGYGATTTRTTRTCWGGA
AgaDef-R	RTTWCKRCAAACRCAAAC
Drosomycin-F	GATTGYTTRTCWGGAAGATATAARG
Drosomycin-R	RCATCCTTCRCACCARCAYTTYAAW
Dro-cecropin-F	GGATGGTTRMGWAARTTRGGAAARA
Dro-cecropin-R	WCKAGCAGTAGCAGCAACRTTAGCA
Metchnikowin-F	CATMGWCATCAAGGACCTATTTTYG
Metchnikowin-R	ATAAATAGGTCCAGGWCKAGGTTGR
PduDef-F	GCTACTTGYGATTTRTTRTCWGCTT
PduDef-R	WCKWCKACAAGTACAAACAGCYTTW
Stomoxyn -F	MGWGGATTYMGWAARCATTTYAAYA
Stomoxyn-R	AGTAGCAGCAACAACAGCAGCTCCW

Letters Y, R, W, K and M, in degenerate primers represent nucleotide mixtures of C/T, A/G, A/T, G/T and A/C, respectively.

### 3D structure analysis of the screened AMP genes

After inferring the genes sequences, the predicted peptide sequences, secondary structure, 3D structure and active sites were analyzed using bioinformation professional analysis websites (http://www.imtech.res.in/raghava/antibp/;http://amp.biosino.org/;http://zhanglab.ccmb.med.umich.edu/I-TASSER/output/S90438/**)**.

### Construction of recombinant expression plasmid

The DNA fragment coding the stomoxynZH1 amino acids (189 bases) was amplified by PCR using the plasmid pMD18-stomoxynZH1 as a template. The forward primer was 5′ -CCGGAATTCAGAGGATTTCGTAAGCA- 3′ containing an *EcoR*I restriction site, and the reverse primer was 5′-AATGCGGCCGCAGTAGCAGCAACAACAGCA-3′ containing a *Not*I restriction site. The amplified fragment was digested with *EcoR*I and *Not*I enzymes, and ligated with the *EcoR*I/*Not*I-linearized pET32a expression vector. The recombinant plasmid was transformed into *E*. *coli* DH5α. Positive clones were identified by colony PCR screening. The positive plasmid was sequenced by TSINGKE Biological Technology Company (Wuhan, China) and then transformed into *E*. *coli* BL21 (DE3).

### Protein expression and purification

The transformed *E*. *coli* BL21 (DE3) cells were cultured for 8 h to exponential phase (OD_600_ = 0.6) in LB medium supplemented with 200 μg/mL ampicillin and then induced with IPTG (1 mM final concentration). The cells were harvested by centrifugation at 8000×g for 10min at 4°C, suspended in 1× phosphate buffer saline (PBS) containing 0.5 mM EDTA /0.5 mM PMSF and then lysed by ultrasonication on ice. The supernatant was collected by centrifugation at 12000×g for 20 min at 4°C and then dialyzed against PBS. The target protein was applied to a nickel ion metal chelating affinity chromatography and then eluted with a gradient concentration of imidazole. The eluted fractions were run on SDS-PAGE (12%) and then detected by Coomassie Brilliant Blue. The protein concentration was determined at 280 nm by a NanoDrop 2000 Spectrophotometer (Thermo, USA).

### Antimicrobial activity assay

The antimicrobial activity of stomoxynZH1 was determined by inhibition-zone assay against Gram-positive bacterium *S*. *aureus*, Gram-negative bacterium *E*. *coli*, fungus *R*. *solani* Khün (rice)-10, and fungus *S*. *sclerotiorum* (Lib.) de Bary-14., as previously described [19]. The bacterial strains were spread on Mueller–Hinton plates, and the pathogenic fungal block was inoculated into potato dextrose agar plates. Metal cylinders 5-mm in diameter were placed on each plate surface, to which either 100μL of purified Trx-stomoxynZH1 fusion proteins or purified Trx fusion protein was added (in duplicate). Ampicillin was used as a positive control in the bacterial strains, and then the plates were incubated at 37°C for 24 h, whereas the fungal strains were incubated for 2–3 days at 28°C. Plates exhibiting zones of growth inhibition were scored as showing antimicrobial activity and photographed against a black background to measure the diameter of the inhibition zone accurately. All experiments were performed in triplicate.

The minimum inhibitory concentration (MIC) in μg/mL for bacterial strains was determined by a standard serial dilution method in sterilized 96-well plates [20]. The MIC of Trx-stomoxynZH1 against both fungi was determined as previously described [21]. In brief, PDA plates were amended with various concentrations of Trx-stomoxynZH1 (0–250 μg/mL). Each plate was inoculated with a mycelial plug of *R*. *solani* Khün (rice)-10 or *S*. *sclerotiorum* (Lib.) de Bary-14. All plates were incubated in triplicate for each concentration at 28°C for 3 days. Plates without any Trx-stomoxynZH1 served as a control. The MIC was defined as the lowest peptide concentration for a complete inhibition of bacterial and fungal growth after the optimum incubation time. Results are presented as the mean values of three independent experiments.

## Results

### Screening and first-strand cDNA cloning of AMP genes

**Fig 1:**
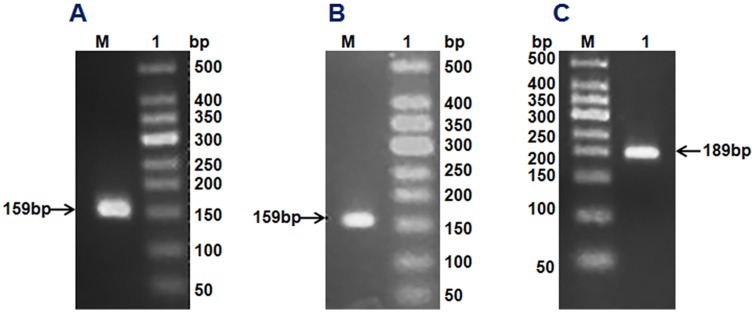
Gel electrophoresis analysis of *H*.*illucens* (L.) antimicrobial gene screening. cecropinZ1 amplification (A). M: 50 DNA Markers; Lane 1: cecropinZ1 PCR product of 159 bp. Sarcotoxin amplification (B). M: 50 DNA Markers; Lane 1: sarcotoxin PCR product of 159 bp. stomoxynZH1 amplification (C). M: 50 DNA Markers; Lane 1: stomoxynZH1 PCR product of 189 bp.

Using the corresponding degenerate primers and first-strand cDNA of *H*. *illucens* (L.) as a template, we obtained DNA fragments of 159, 159 and 189 bp by gradient PCR amplification, (Fig 1) and found that these fragments belong to cecropin, sarcotoxin and stomoxyn, respectively. These DNA fragments were ligated into the pMD18-T vector and then transformed into *E*. *coli* DH5α. Positive clones from each ligation were selected for sequencing. Seven genes were screened on the basis of the amino acid sequences. Four isoforms were detected for sarcotoxin [sarcotoxin 1, sarcotoxin (2a), sarcotoxin(2b), and sarcotoxin 3] whereas only one form was found for cecropin (cecropinZ1). Meanwhile, two isoforms were screened for stomoxyn [stomoxynZH1 and stomoxynZH1 (a)].

Alignment of mature peptides of AMP genes was identified in this study, and the original counterpart AMP sequences revealed small identity to these peptides. Table 3 summarizes the screened gene sequences, gene alignment results, and their optimum annealing temperatures.

**Table 3:** Summary of AMP screened genes in *H*.*illucens* (L.).

Sequence name	Size (aa)	Best Tm (°C)	Similarity (%)	Remarks
Sarcotoxin1	53	54.8	44.44	--
Sarcotoxin(2a)	53	54.8	46.29	99% similarity with sarcotoxin1; different in base No.26,57
Sarcotoxin(2b)	53	54.8	46.29	98% similarity with sarcotoxin1; different in base No.26,57,59
Sarcotoxin3	47	54.8	42.55	--
CecropinZ1	53	56.8	46.29	--
StomoxynZH1	63	50.6	46.29	--
StomoxynZH1(a)	63	50.6	46.29	99% similarity with Stomoxyn ZH1; different in base No.21

### 3D structures of the AMP genes isolated from *H*. *illucens* (L.)

**Fig 2:**
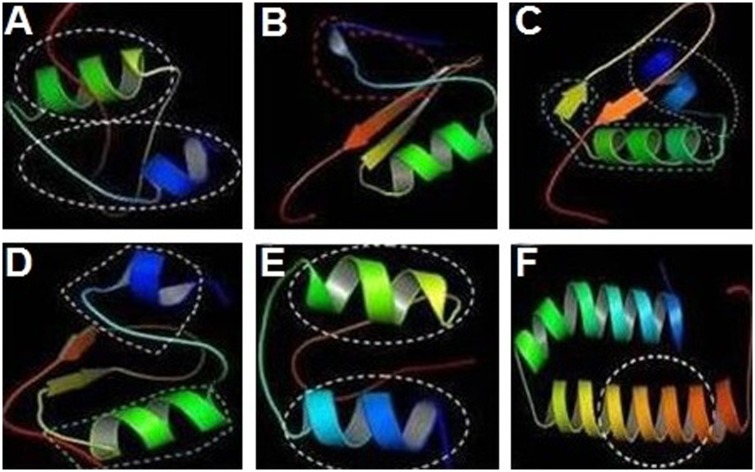
3D structures of the AMP genes isolated from *H*. *illucens* (L.). CecropinZ1 (A). Sarcotoxin1 (B). Sarcotoxin(2a) (C). Sarcotoxin(2b) (D). Sarcotoxin3 (E). StomoxynZH1 and StomoxynZH1(a) (F).

Bioinformatics analysis revealed that cecropinZ1 is composed of a double α-helix, which serves mainly as its activity area, as illustrated inside the circle part; the double α-helix comprises four active sites and two active regions (Fig 2A). The 3D structure of sarcotoxin1 reveals that it is composed of a α-helix, a β-fold, and an incomplete α-helix, which act as the active areas (Fig 2B). The 3D structure of sarcotoxin(2a) confirms the presence of a double α-helix and a β-fold, and the activity areas are located within the double α-helix and part of the β-fold, as described inside the circle (Fig 2C). The 3D structure of sarcotoxin(2b) is composed of a double α-helix and a β-fold, and the active areas are in the double α-helix, as illustrated inside the circle (Fig 2D). Meanwhile, the 3D structure of sarcotoxin3 contains only a double α-helix, which acts as the active area (Fig 2E). The 3D structure of stomoxynZH1 and stomoxynZH1 (a) is composed of a double α-helix, which serves as the active area (Fig 2F). S1 file shows the sequences of these genes and their corresponding amino acids.

### Construction and identification of recombinant expression plasmid

**Fig 3:**
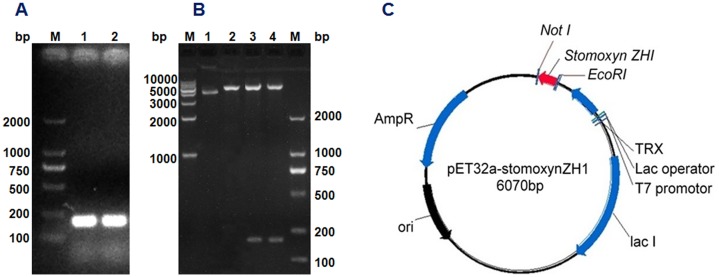
Reconstruction of recombinant plasmid pET32a-stomoxynZH1. StomoxynZH1 double digestion (A). M: 2000 bp DNA Markers; Lanes 1 and 2: stomoxynZH1 PCR digested with *EcoR*I and *Not*I. Double digestion of pET32a with *EcoR*I/*Not*I and restriction enzyme analysis of pET32a-stomoxynZH1 expression plasmid (B). M: 2000 bp and 1 kb DNA Markers; lane 1: undigested plasmid, lane 2: pET32a digested with *EcoR*I/*Not*I, lanes 3 and 4: pET32a-stomoxynZH1 construct digested with *EcoR*I and *Not*I. Schematic of the pET32a-stomoxynZH1 expression vector used in this study (C).

The stomoxynZH1 PCR product with a length of 189 bp (Fig 3A) was cloned into the pET32a expression vector to produce a construct encoding N-terminally tagged his 6-thioredoxin-stomoxynZH1. The constructed plasmid designated as pET32a-stomoxynZH1 was analyzed using *EcoR*I and *Not* I (Fig 3B) and then sequenced. The restriction enzyme analysis result and DNA sequence analysis confirmed that the sequence of the anticipated 189 bp stomoxynZH1 gene was accurate and correctly inserted into the pET32a expression vector. Fig 3C illustrates the pET32a-stomoxynZH1 recombinant plasmid.

### Recombinant expression and purification of the thioredoxin-stomoxynZH1 (Trx-stomoxynZH1) in *E*. *coli*

**Fig 4:**
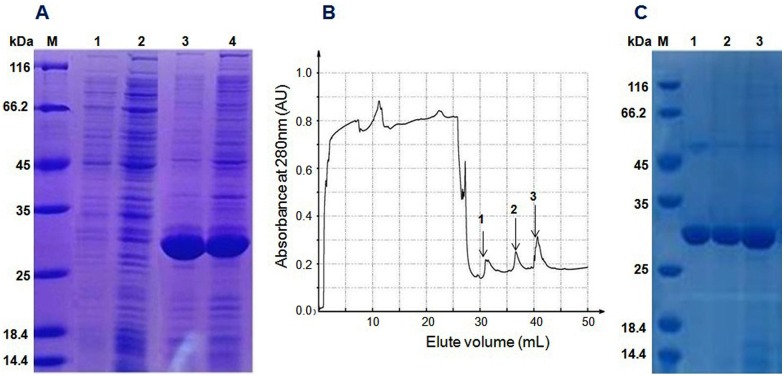
Expression of the stomoxynZH1 gene in *E*.*coli* and purification of Trx- stomoxynZH1. 12% SDS-PAGE analysis of the recombinant protein expressed in BL21 (DE3) (A). M: protein standard molecular weight marker, lanes 1 and 2 are samples collected before induction with IPTG, lanes 3 and 4 are samples collected after induction with IPTG. Chromatography curve recorded by the Nanjing Puyang Scientific Instrument Research Institution Purifier system (B). Arrows 1, 2 and 3 are the fractions of Trx-stomoxynZH1 purified by the nickel ion metal chelating affinity chromatography. SDS-PAGE (12%) analysis of Trx-stomoxynZH1 purification (C). M, protein marker; lanes 1–3 are the purified fractions of Trx-stomoxynZH1.

The Trx-stomoxynZH1 fusion protein was expressed in *E*. *coli* BL21 (DE3) by using the pET32a vector. The expression of this fusion protein was confirmed by the appearance of a 30-kDa protein band in the SDS-PAGE gel and was consistent with the predicted molecular weight. The maximum production of the fusion protein was achieved within 8h at 28°C (Fig 4A) without any apparent toxicity to the bacteria. The Trx-stomoxynZH1 fusion protein was applied to affinity chromatography utilizing the metal-binding polyhistidine (His-tag), after which high purity purified protein was obtained (Fig 4B and 4C). The concentration of purified Trx-stomoxynZH1 was measured by absorbance at 280 nm, and ~ 1.85 mg/mL fusion protein was obtained.

### Antimicrobial assay

**Fig 5:**
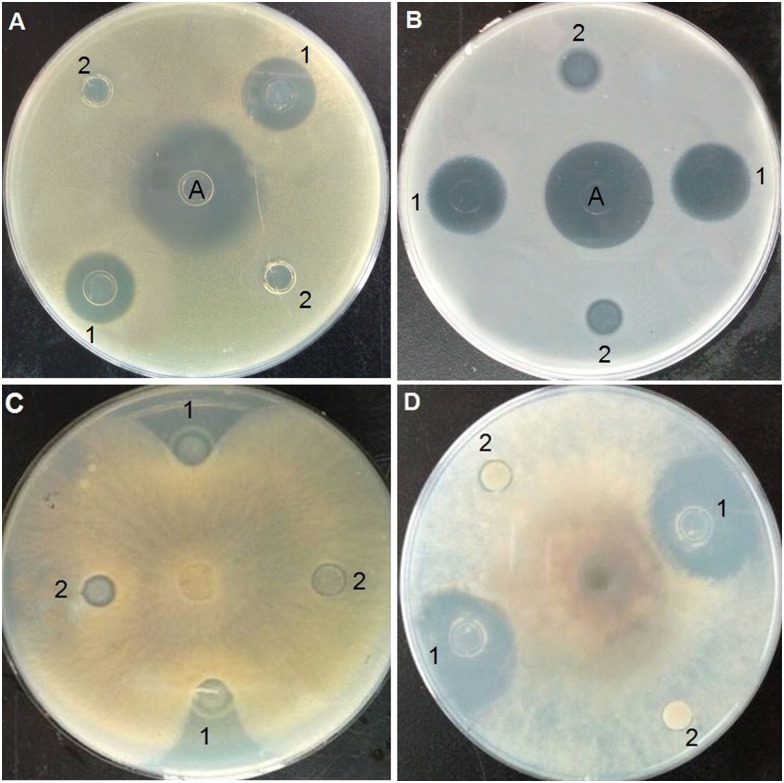
Inhibition zone test of purified Trx-stomoxynZH1 against bacterial and fungal strains. *S*.*aureus* (A). *E*.*coli* (B). *R*. *solani* Khün(rice)-10 (C), and *S*. *sclerotiorum* (Lib.) de Bary-14 (D). A: ampicillin (1 μg/mL), 1 is purified Trx-stomoxynZH1 fusion protein, and 2 is purified Trx fusion protein (negative control).

Inhibition-zone and minimal inhibitory concentration (MIC) assays were carried out against Gram-positive bacteria, Gram-negative bacteria, and fungi to evaluate the antimicrobial activity of purified Trx-stomoxynZH1. As shown in Fig 5, Trx-stomoxynZH1 exerted strong inhibitory effects against *S*. *aureus* (Fig 5A), *E*. *coli* (Fig 5B), *R*. *solani* Khün (rice)-10 (Fig 5C), and *S*. *sclerotiorum* (Lib.) de Bary-14 (Fig 5D). MIC results revealed that Trx-stomoxynZH1 proteins exhibited potent antimicrobial activities against all tested pathogens. The thioredoxin alone did not display activity even at the highest concentration. The MICs of Trx-stomoxynZH1 required to eliminate the growth of bacterial and fungal strains are summarized in Table 4.

**Table 4:** Minimal inhibition concentration (MIC) of Trx-stomoxynZH1.

Microorganism Strain	MIC (μg/mL)
*E*. *coli*	15–30
*S*. *aureus*	27–54
*R*. *solani* Khün (rice)-10	>98
*S*. *sclerotiorum (Lib*.*) de Bary-14*	>98

## Discussion

Upon activation of the insects’ innate immune system, a wide range of effectors molecules including AMPs, are produced [10]. AMPs are ideal alternatives to conventional antibiotics because of their many desirable features, such as low-cost and rapid production, heat-tolerance, relatively broad antimicrobial spectrum, and low toxicity to eukaryotic cells [22, 23]. Much research focused on AMPs from dipteral insects, such as tsetse fly [24], flesh fly [25], fruit fly [26], and house fly [27].

Although *H*. *illucens* (L) populations are susceptible to many infectious agents, including bacteria and parasites, studies on their antimicrobial active compounds are rare. In the current study, a comprehensive screening of AMP genes from *H*. *illucens* (L.) was conducted to explore relatively new AMP genes. Seven gene fragments of three types of AMPs were acquired. Gene sequence alignment and prediction of the protein structures of these amplified fragments revealed that these peptides are AMPs in *H*. *illucens* (L.) with lower sequence similarity compared with the AMPs from other dipterans insects.

Since the discovery of cecropin in *Hyalophora cecropia* [28], numerous cecropin family genes have been isolated and characterized from different dipteral insects [26, 29] and other organisms, including mammals [30]. Insect cecropins are small cationic polypeptides located in the hemolymph that exhibit a broad spectrum of activities against fungi, bacteria, viruses, and parasites [31]. The present study is the first to report on the isolation of a new cecropin gene from *H*. *illucens* (L.). A comparative bioinformatics analysis using different bioinformation professional analysis websites revealed that, the current cecropin has two active sites (N-myristoylation site and protein kinase C phosphorylation site) identical to those in cecropin in *H*. *cecropia* [28] and *SibaCec* from black fly [32]. The cecropin isolated in the present study has conserved consensus sequences in starting and terminal regions similar to those isolated from other dipterans, such as *Drosophila virilis*, *Drosophila melanogaster*, *Sarcophaga pergrina*, and *C*. *capitates* [33].

Sarcotoxins belong to cecropins, the most abundant family of insect origin linear AMPs [34]. Compared with the original sarcotoxin subdivided into three isoforms, namely, sarcotoxin I, II, and III [35], the amplified sarcotoxin obtained in this study has four isoforms. Three of these isoforms consist of 53 amino acid residues and differ in only one amino acid residue; the fourth one is composed of 47 amino acids. The composition of sarcotoxin isoforms varies in sarcotoxin1 of the flesh fly, whereas it is a mixture of three similar proteins each with 39 amino acid residues [36]. Striking similarities can be observed between parts of the amino- and carboxy-terminals of these molecules and sarcotoxin IA [37], sarcotoxin IC [38], sarcotoxin Pd [34], and sarcotoxin IB [39]. Active sites that are not present in the original sarcotoxin have also been obtained, including three N-myristoylation sites [sarcotoxin1]; extra amidation sites and three additional N-myristoylation sites [sarcotoxin(2a), sarcotoxin(2b)]; an additional N-glycosylation site, an additional N-myristoylation site, and an additional casein kinase II phosphorylation site [sarcotoxin3]; this finding suggests the different functions of the amplified sarcotoxin from the original sarcotoxin [35, 40, 41]. The sarcotoxin N-terminus Gly1-Trp2 is a requirement for the binding of sarcotoxin-IA to the lipid A of LPS, suggesting that the N-terminus Gly1-Trp2 is important for the activity against *E*. *coli* [31]. Basing on these findings, we hypothesized that the current sarcotoxin exerts a bactericidal effect against Gram-negative bacteria.

Stomoxyn is a linear cysteine-free insect AMP that is composed of a 42 amino acid residue and was first isolated from the stable fly *Stomoxys calcitrans* [42]. The present study reports the identification of a novel AMP, stomoxynZH, in black solider fly larvae. The putative mature stomoxynZH had 60 amino acids with a molecular weight of 6.1 kDa. Although a α-helix structure is found in the AMPs of most insects [31], the 3D structure predicts that stomoxynZH adopts a α-helix structure similar to that of stomoxyn, an antibacterial and antifungal peptide forming a α-helical structure in phospholipid membrane mimic organic compounds [43]. The deduced amino acid sequence showed significant similarity with insect stomoxyn from *S*. *calcitrans* (45%) and LSerStomox 1 and 2 from blow fly (30%). Multiple alignments revealed that stomoxynZH shared some conservation locations of the mature peptide sequence with stomoxyn from other insects, particularly the amino- and carboxy-terminals. Although the two stomoxyn forms screened in this study did not exhibit the two active sites located in the original sequence, these stomoxyn forms showed three other active areas.

While stomoxyn, sarcotoxin, and cecropin belong to the same antimicrobial superfamily (Cecropins) [31], limited research compared the antimicrobial effects of stomoxyn with those of sarcotoxin and cecropin. Moreover, stomoxyn has a broad activity spectrum affecting the growth of microorganisms, with low toxicity to eukaryotic cells and apparent activity within a few minutes of exposure [44]. In consideration of these observations, stomoxyn was selected for large-scale production to allow the investigation of its antimicrobial spectrum.

The heterologous expression of recombinant proteins is a cost-effective and scalable method for the commercial production of AMPs [45]. Several studies expressed insect AMPs in *E*. *coli* including cecropin from *Plutella xylostella* [45], defensin-like antifungal peptide from the whitefly *Bemisia tabaci* [46], and defensin-like peptides from *Protaetia brevitarsis seulensis* [5]. However, recombinant AMPs may affect the host viability. Hence, the pET32a-stomoxynZH1 expression vector was used to prevent the toxicity of the recombinant stomoxynZh1 to the host cells and obtain high quantities of soluble protein. In the expression vector, the TRX protein was stable, and a highly soluble partner was fused with the target peptide. As expected, the resulting recombinant Trx-stomoxynZH1 was highly expressed in *E*. *coli* BL21, and the soluble component was obtained. The expression of AMPs as a thioredoxin fusion protein enhances protein quantity and solubility [47]. Numerous studies successfully obtained recombinant AMPs using the Trx-fusion system [46, 47].

In the current study, the purified recombinant Trx-stomoxynZH1 exhibited evident antimicrobial activities against *E*. *coli*, *S*. *aureus*, *R*. *solani* Khün (rice)-10, and *S*. *sclerotiorum* (Lib.) de Bary-14. This finding revealed that the peptide displays characteristic activities against gram-negative bacteria, gram-positive bacteria, and filamentous fungi. This result conforms to earlier findings that stomoxyn from *S*. *calcitrans* possesses potent activity against commonly tested bacterial and filamentous fungal strains [43]. Antimicrobial assay revealed that the expressed Trx-stomoxynZH1 demonstrated strong activity against *E*. *coli*, whereas *S*. *aureus* was less sensitive. These results were similar to those of stomoxyn from another dipteran, *Lucilia sericata* [48]. In the previous report, the defensin-like antimicrobial peptide *Psdefensin* from *Protaetia brevitarsis seulensis* was cloned into pMAL expression system in an approach similar to that of this study. The purified MBP-Psdefensin proteins exhibited moderate antimicrobial activities against both Gram-negative bacteria and Gram-positive bacteria [5].

MIC data analysis revealed that the fungicidal property of stomoxynZH1 against *R*. *solani* Khün (rice)-10 was similar to the effectiveness of stomoxynZH1 against *S*. *sclerotiorum* (Lib.) de Bary-14. Alem et al. [49] were able to express the naturally derived antimicrobial peptide AqAMP from *Amaranthus quitensis*, by pET32a (+) in *E*. *coli*. They concluded that Trx-AqAMP had in vitro antifungal activity against a set of phytopathogens such as *A*. *solani*, *F*. *oxysporum* f. sp. *Lycopersici and P*. *digitatum*. Other linear AMPs effectively inhibit fungal growth; cecropin B and magainin II display strong antifungal activities against *R*. *solani* and *Fusarium oxysporum*, respectively [50]. These observations suggest that α-helices may be associated to the linear antimicrobial activity against fungi.

The identified peptide with a wide-spectrum antimicrobial activity may be beneficial in the treatment of pathogenic diseases. To ensure the applicability of AMPs, they should combine certain desired properties such as, high sensitivity and specificity for target pathogens, low toxicity for plant and human cells, biodegradability, low possibility for resistance development by the pathogen population and tolerance to proteases as well as to changes of temperatures and pH. While the AMPs behave differently than classical synthetic antibiotic, further studies are needed to simulate the environment of AMP application and to explore a novel approach for AMP application from various origins.

## Conclusion

In the current study, seven new gene fragments of three types of AMP genes were acquired from *H*. *illucens* (L.). The DNA sequence alignment and forecast-analysis of protein structure revealed that these seven AMP genes were less similar to other AMP genes. StomoxynZH1 was expressed as a fusion protein with thioredoxin in bacteria. To the best of our knowledge; this study is the first to use heterologous expression technology to produce an AMP gene from the black solider fly. Thioredoxin mediating the fusion expression of stomoxynZH allowed the peptide expression to be quick, easy, and beneficial for large-scale stomoxynZH production. Moreover, the purified Trx-stomoxynZH1 was tested against a set of pathogenic microorganisms; results revealed that Trx-stomoxynZH1 has a broad activity spectrum affecting the growth of gram-negative bacteria, gram-positive bacteria, and filamentous fungi.

Thus, Trx-stomoxynZH1 may be used not only in the treatment of human and animal diseases but also in the development of plant resistance against pathogens. However to be considered as a potential biomolecules, additional detailed studies, such as in vivo testing of its antimicrobial activity, are required.

## Supporting Information

S1 File Identified genes Sequences and their corresponding amino acids. (DOCX)
