# Supplementary material for: Screening, Expression, Purification and Functional Characterization of Novel Antimicrobial Peptide Genes from Hermetia illucens (L.)
Source: PLoS One. 2017 Jan 5;12(1):e0169582. doi: 10.1371/journal.pone.0169582 (PMC5215879; doi:10.1371/journal.pone.0169582)
Supplement: S1 File — (DOCX) [file pone.0169582.s001.docx]

**Appendix 1:**

**Nucleotide sequence of Sarcotoxin1:**

ggatggttgaagagaaagattggaatgaaattcattttgggaacaactttggccattgtc 60

gttgccatctttggccaatgtcaagctgccacctggagttataatccaaatggtggtgct 120

accgtaacatggactgctaacgttgctgctactgctaga 159

**Amino acid sequence of Sarcotoxin1:**

Gly Trp Leu Lys Arg Lys Ile Gly Met Lys Phe Ile Leu Gly Thr 15

Thr Leu Ala Ile Val Val Ala Ile Phe Gly Gln Cys Gln Ala Ala 30

Thr Trp Ser Tyr Asn Pro Asn Gly Gly Ala Thr Val Thr Trp Thr 45

Ala Asn Val Ala Ala Thr Ala Arg 53

**Nucleotide sequence of Sarcotoxin(2a):**

ggatggttgaagagaaagattggaaagaaattcattttgggaacaactttggccatcgtc 60

gttgccatctttggccaatgtcaagctgccacctggagttataatccaaatggtggtgct 120

accgtaacatggactgctaacgttgctgctactgctaga 159

**Amino acid sequence of Sarcotoxin(2a):**

Gly Trp Leu Lys Arg Lys Ile Gly Lys Lys Phe Ile Leu Gly Thr 15

Thr Leu Ala Ile Val Val Ala Ile Phe Gly Gln Cys Gln Ala Ala 30

Thr Trp Ser Tyr Asn Pro Asn Gly Gly Ala Thr Val Thr Trp Thr 45

Ala Asn Val Ala Ala Thr Ala Arg 53

**Nucleotide sequence of Sarcotoxin(2b):**

ggatggttgaagagaaagattggaaagaaattcattttgggaacaactttggccatcgcc 60

gttgccatctttggccaatgtcaagctgccacctggagttataatccaaatggtggtgct 120

accgtaacatggactgctaacgttgctgctactgctaga 159

**Amino acid sequence of Sarcotoxin(2b):**

Gly Trp Leu Lys Arg Lys Ile Gly Lys Lys Phe Ile Leu Gly Thr 15

Thr Leu Ala Ile Ala Val Ala Ile Phe Gly Gln Cys Gln Ala Ala 30

Thr Trp Ser Tyr Asn Pro Asn Gly Gly Ala Thr Val Thr Trp Thr 45

Ala Asn Val Ala Ala Thr Ala Arg 53

**Nucleotide sequence of Sarcotoxin3:**

ggatggttga agagaaagat tggaatgatg atgaagaact ccaacttcaa cagtactgaa 60

gaacgagaag ctgcgaagaa gaattacaaa aggaaatacg ttccttggtt ctctggtgct 120

aacgttgctg ctactgctag a 141

**Amino acid sequence of Sarcotoxin3:**

Gly Trp Leu Lys Arg Lys Ile Gly Met Met Met Lys Asn Ser Asn 15

Phe Asn Ser Thr Glu Glu Arg Glu Ala Ala Lys Lys Asn Tyr Lys 30

Arg Lys Tyr Val Pro Trp Phe Ser Gly Ala Asn Val Ala Ala Thr 45

Ala Arg 47

**Nucleotide sequence of Cecropin:**

ggatggttga aaaagattgg aaagatgaaa ttcattttgg gaacaacttt ggccattgtc 60

attgccattt ttggccaatg ccaagctgcc acctggagtt ataatccaaa tggtggtgct 120

accgtaacatggactgctaacgttgctgctactgctaga 159

**Amino acid sequence of Cecropin:**

Gly Trp Leu Lys Lys Ile Gly Lys Met Lys Phe Ile Leu Gly Thr 15

Thr Leu Ala Ile Val Ile Ala Ile Phe Gly Gln Cys Gln Ala Ala 30

Thr Trp Ser Tyr Asn Pro Asn Gly Gly Ala Thr Val Thr Trp Thr 45

Ala Asn Val Ala Ala Thr Ala Arg 53

**Nucleotide sequence of StomoxynZH1:**

agaggatttc gtaagcattt caacaactta ccaatctgcg tggaaggatt agctggagat 60

attggttcca ttcttcttgg tgttgaatca gatatcggtg cattggctgg tgccatcgcc 120

aatttggctc ttatcgctgg tgaatgcgct gcacaaggtg aagcaggagc tgctgttgtt 180

gctgctact 189

**Nucleotide sequence of StomoxynZH1(a):**

agaggatttcgtaagcattttaacaacttaccaatctgcgtggaaggattagctggagat 60

attggttccattcttcttggtgttgaatcagatatcggtgcattggctggtgccatcgcc 120

aatttggctcttatcgctggtgaatgcgctgcacaaggtgaagcaggagctgctgttgtt 180

gctgctact 189

**Amino acid sequence of Stomoxyn & StomoxynZH1(a):**

Arg Gly Phe Arg Lys His Phe Asn Asn Leu Pro Ile Cys Val Glu 15

Gly Leu Ala Gly Asp Ile Gly Ser Ile Leu Leu Gly Val Glu Ser 30

Asp Ile Gly Ala Leu Ala Gly Ala Ile Ala Asn Leu Ala Leu Ile 45

Ala Gly Glu Cys Ala Ala Gln Gly Glu Ala Gly Ala Ala Val Val 60

Ala Ala Thr 63
